# Adiponectin Reverses the Proliferative Effects of Estradiol and IGF-1 in Human Epithelial Ovarian Cancer Cells by Downregulating the Expression of Their Receptors

**DOI:** 10.1007/s12672-018-0331-z

**Published:** 2018-03-30

**Authors:** Marta Hoffmann, Justyna Gogola, Anna Ptak

**Affiliations:** Department of Physiology and Toxicology of Reproduction, Chair of Animal Physiology, Institute of Zoology and Biomedical Research, Jagiellonian University, Krakow, Poland

**Keywords:** Epithelial Ovarian Cancer Cells, Epithelial Ovarian Cancer Cell Lines, AdipoR1 Expression, Adipate, Granulosa Tumor Cell Line

## Abstract

The expression of adiponectin receptors AdipoR1 and AdipoR2 has been reported in the human ovary and ovarian cancer tissues. Moreover, adiponectin has been reported to act as an anti-tumor factor by inhibiting cancer cell proliferation. Thus, we investigate whether adiponectin and its receptors influence ovarian cancer development. In the present study, we found that adiponectin was not expressed in the granulosa cell line (COV434), and epithelial ovarian cancer cell lines (OVCAR-3, SKOV-3, and Caov-3). Additionally, we found that AdipoR1 and AdipoR2 expression is lower in epithelial ovarian cancer cells than in granulosa tumor cells. Endogenous 17β-estradiol as well as exogenous estrogens, such as bisphenol A and its chlorinated and brominated analogs do not affect adiponectin receptor expression. We found that adiponectin inhibited the growth of OVCAR-3 and SKOV-3 cells, and that this effect was independent of apoptosis. Moreover, adiponectin reverses the stimulatory effects of 17β-estradiol and insulin-like growth factor 1 on cell proliferation by downregulating the expression of their receptors, whereas progesterone increased the sensitivity of cancer cells to adiponectin by upregulating AdipoR1 and AdipoR2 expression. These results suggest interactions between adiponectin and various ovarian steroid hormone and growth factor pathways in ovarian cancer cells.

## Introduction

Ovarian cancer remains the leading cause of death among women, with an estimated 150,000 annual deaths worldwide [1]. Due to its non-specific symptoms, most cases of ovarian cancer are detected when the disease has advanced to a late stage that associates with poor survival. Thus, approaches that would increase its early detection are urgently needed to reduce mortality. Ovarian cancer can be classified into three types based on the cell of its origin, namely, epithelial, stromal, and germ, with each type conferring different histopathological features and clinical outcomes [2]. Epithelial ovarian cancer is the most common ovarian malignancy; it originates in epithelial cells found on the surface of the ovary and accounts for ~ 80–90% of ovarian malignancies. Stromal tumors, on the other hand, account for ~ 7% of ovarian malignancies, and the most frequently diagnosed stromal tumor type is the granulosa cell tumor (GCT).

There is emerging evidence to indicate that obesity is the main independent risk factor for ovarian cancer [3–5]. Although the correlation between ovarian cancer and obesity has been linked to hormones, it is not clear how they can trigger cancer in obese women. Hormones and growth factors have important roles in regulating cell proliferation, differentiation, and apoptosis. For example, 17β-estradiol (E2), progesterone (P4), and insulin-like growth factor 1 (IGF-1) have all been proposed to influence ovarian cancer development [6, 7]. Adipokines, hormones secreted from adipose tissues that may promote obesity, may also affect cancer development.

Adiponectin, an adipokine with a molecular weight of 30 kDa, is found in the serum, where it exists in four isoforms, namely, trimeric (90 kDa), hexameric (180 kDa), and high-molecular-weight (360 and 400 kDa) isoforms [8]. At a serum concentration of 5–30 μg/ml, it is the most abundant circulating peptide hormone. In obese adults, however, the serum adiponectin level is reduced [9]. Adiponectin has been reported to act as an anti-tumor factor by inhibiting cancer cell proliferation [10, 11]. Other studies report a role for adiponectin in obesity-associated cancer such as those of the breast, cervix, and endometrium. However, the role of adiponectin in ovarian cancer has been studied much less. For example, Jin et al. reported that adiponectin levels were significantly lower in ovarian cancer patients than in healthy individuals, but the reason for this is not clear [12]. Furthermore, the biological actions of adiponectin are mediated through interactions with its receptor subtypes, AdipoR1 and AdipoR2. Li et al. showed that a low AdipoR1 expression level in cancerous ovarian tissues serves as an independent prognostic indicator of the disease [13]. In the human granulosa KGN cell line, AdipoR1 functions in cell survival, whereas AdipoR2 regulates steroid production [14].

Several endogenous, as well as exogenous factors, including insulin, thiazolidinediones, metformin, and bisphenol A (BPA), can regulate the production and secretion of adiponectin in the 3T3-L1 adipocyte cell line [15–18]. On the other hand, several lines of evidence indicate that endocrine disrupting chemicals, such as BPA, can induce obesity [19, 20]. BPA, a commercial product commonly used in polycarbonate plastics and epoxy resins [21], possesses estrogenic activity and promotes ovarian cancer cell proliferation [22, 23] and migration [24]. Epidemiological studies report that humans have detectable serum levels of not only BPA, but also its halogenated derivatives, tetrabromobisphenol A (TBBPA) and tetrachlorobisphenol A (TCBPA) [25–27].

We aimed to investigate whether adiponectin and its receptors, AdipoR1 and AdipoR2, are expressed in human epithelial ovarian cancer cell lines. We also examined whether BPA and its analogs can affect the expression of adiponectin and its receptors in ovarian cancer cells. The effects of adiponectin on cell proliferation and apoptosis were also examined. Finally, we investigated whether E2, P4, and IGF-1 can regulate AdipoR1 and AdipoR2 expression and modulate the effects of adiponectin on the proliferation of ovarian cancer cells.

## Materials and Methods

### Cell Culture and Chemicals

OVCAR-3, SKOV-3, and Caov-3 were purchased from the American Type Culture Collection (Manassas, VA, USA). COV434 cells were obtained from the European Collection of Authenticated Cell Cultures (ECACC, Sigma-Aldrich, St. Louis, MO, USA). The human ovarian serous carcinoma cell lines OVCAR-3, SKOV-3, and Caov-3 were cultured in RPMI 1640, McCoy’s, and Dulbecco’s modified Eagle’s medium (DMEM), respectively. Media were supplemented with 10% fetal bovine serum (FBS) (Thermo Fisher Scientific Inc., Carlsbad, CA, USA). The human metastatic granulosa cell line COV434 was cultured in DMEM supplemented with 2 mM l-glutamine (Biowest, Nuaillé, France) and 10% FBS. All cell cultures were maintained at 37 °C in a humidified atmosphere containing 5% CO_2_.

Human adiponectin, 17β-estradiol, progesterone, and insulin-like growth factor 1 were purchased from Sigma-Aldrich. BPA (AccuStandard, New Haven, CT, USA) and E2 were dissolved in absolute ethanol. TBBPA and TCBPA were purchased from Santa Cruz Biotechnology (Santa Cruz, CA, USA) and dissolved in DMSO. The final concentration of ethanol and DMSO in the medium was 0.1%. Viability of cells was not affected at this concentration.

### Real-Time PCR Analysis

COV434, OVCAR-3, SKOV-3, and Caov-3 cells were seeded in 96-well plates and cultured until 70% confluent in the indicated media for 24 h. The culture medium was then removed, and the cells were rinsed with PBS and stored at − 20 °C. To investigate the effects of E2, P4, IGF-1, BPA, TBBPA, and TCBPA on the expression of AdipoR1 and AdipoR2, the culture medium was replaced with fresh medium 24 h before the cells were treated with compounds. The cells were then exposed to vehicle (medium, 0.1% DMSO, or 0.1% ethanol), E2 (10 nM), P4 (100 μM), IGF-1 (100 ng/ml), or BPA, TBBPA, or TCBPA (1, 10, and 100 nM) for 24 h. To investigate the effects of adiponectin on the expression of ERα, ERβ, PR, and IGF1R, the culture medium was replaced with fresh medium 24 h before the cells were treated with adiponectin at 25 μg/ml for 24 h.

Total RNA was isolated from control and treated cells, followed by cDNA synthesis using the TaqMan Gene Expression Cells-to-CT Kit (Applied Biosystems, Foster City, CA, USA), according to the manufacturer’s instructions. The lysis solution contained DNase I to digest contaminating genomic DNA. The resulting cDNA was analyzed by real-time PCR using a StepOnePlus Real-Time PCR System (Applied Biosystems) and TaqMan Gene Expression Assays together with a TaqMan Gene Expression Master Mix and ROX Reference Dye (Applied Biosystems). The thermal cycling conditions were as follows: 50 °C for 2 min and 95 °C for 10 min, followed by 40 cycles of 95 °C for 15 s and 60 °C for 60 s. Duplicate control cDNA-free samples were prepared for each gene. The TaqMan Gene Expression Assays used for real-time PCR were as follows: adiponectin [*ADIPOQ*, assay nos. Hs00605917_m1 and Hs00977214_m1], AdipoR1 [*ADIPOR1*, assay no. Hs01114951_m1], AdipoR2 [*ADIPOR2*, assay no. Hs00226105_m1], ERα [*ESR1*, assay no. Hs00174860_m1], ERβ [*ESR2*, assay no. Hs001100353_m1], PR [*PGR*, assay no. Hs01556702_m1], and IGF1R [*IGF1R*, assay no. Hs00609566_m1]. Expression levels were normalized to that of *GADPH* (assay no. 4310884E), and the relative expression was quantified using the 2^−ΔΔCt^ method [28].

### Western Blot Analysis

COV434, OVCAR-3, and SKOV-3 cells were seeded in 24-well plates and cultured until 70% confluent in the indicated media for 24 h. The cells were then removed from plates, transferred to ice-cold lysis buffer, and stored at − 20 °C. The protein concentrations of the lysates were determined by the Bradford method (Bio-Rad Protein Assay, Bio-Rad Laboratories, Hercules, CA, USA). An equal amount of protein (60 μg) from each sample was assayed. Proteins were separated by 10% SDS-PAGE, and then transferred to polyvinylidene fluoride (PVDF) microporous membranes (Millipore, Billerica, MA, USA) using the Bio-Rad Mini-Protean 3 System. The membranes were blocked for 1 h in 0.02 M Tris-buffered saline containing 5% bovine serum albumin and 0.1% Tween 20, and then incubated overnight at 4 °C with antibodies specific for human AdipoR1 (cat. no. sc-46749, Santa Cruz Biotechnology), human AdipoR2 (cat. no. sc-46751, Santa Cruz Biotechnology), ERα (cat. no. sc-542, Santa Cruz Biotechnology), PR (cat. no. sc-539, Santa Cruz Biotechnology), IGF1R (cat. no. ab39675, Abcam, Cambridge, UK), and PARP (cat. no. #9542, Cell Signaling Technology). The membranes were then washed three times in Tris-buffered saline containing 0.1% Tween 20 (TBST) and incubated for 1 h at room temperature with a species-compatible horseradish peroxidase-conjugated secondary antibody (cat. no. sc-2020, Santa Cruz Biotechnology). β-Actin (cat. no. A5316, Sigma-Aldrich) was used as the loading control. Immunopositive bands were visualized using WesternBright Quantum HRP Substrate (cat. no. K-12043 D20, Advansta Inc., Menlo Park, CA, USA). Quantification of protein bands was performed by densitometry using VisionWorks LS Acquisition and Analysis software (UVP, LLC Upland, CA).

### Cell Proliferation

Cell proliferation was measured using AlamarBlue Cell Viability Reagent (Invitrogen, Paisley, UK), according to the manufacturer’s instructions. OVCAR-3 and SKOV-3 cells were exposed to adiponectin (0.025, 0.25, 2.5, or 25 μg/ml) for 48 h. In co-treatment experiments, OVCAR-3 cells were treated with adiponectin (25 μg/ml) and E2 (10 nM), P4 (100 μM), or IGF-1 (100 ng/ml) for an additional 48 h. The AlamarBlue stock solution was aseptically added to the wells after 48 h of culture in an amount equaling 10% of the volume of culture medium. The reduction of resazurin to resorufin was determined after 4 h of incubation by measuring the fluorescence at an excitation wavelength of 560 nm and an emission wavelength of 590 nm using an FLx800 fluorescence microplate reader (BioTek Instruments, Winooski, VT, USA). Data were analyzed using KC JUNIOR Software (BioTek Instruments).

### Caspase-3 Activity

Twenty-four hours before each experiment, the culture medium was replaced with serum-free medium. Treatments consisted of vehicle or adiponectin (0.025, 0.25, 2.5, or 25 μg/ml) for 24 h. The medium was then removed, and the plates were stored at − 70 °C. Cells were lysed in caspase assay buffer (50 mM HEPES, pH 7.4, 100 mM NaCl, 0.1% CHAPS, 1 mM EDTA, 10% glycerol, and 10 mM DTT). The protein concentrations of the lysates were determined by the Bradford method (Bio-Rad Protein Assay). An equal amount of the cytosolic extract (100 μg of protein) from each sample was analyzed. The assay was carried out by adding 100 μM Ac-DEVDAMC (Sigma-Aldrich) and incubating the plates at 37 °C. The amount of fluorescent product was monitored continuously for 120 min with a spectrofluorometer (FLx800 BioTek Instruments) at an excitation wavelength of 355 nm and an emission wavelength of 460 nm. Data were analyzed using KC JUNIOR Software, and normalized to fluorescence levels in vehicle-treated cells.

### Statistical Analysis

Statistical data are presented as means ± SD of three independent experiments performed in triplicate. Statistical analysis was carried out using one-way or two-way ANOVA, followed by Tukey’s test (GraphPad Software, La Jolla, CA, USA). The level of significance was set at *p* < 0.05.

## Results

### Expression of Adiponectin and Its Receptors in Epithelial Ovarian Cancer Cell Lines

**Fig. 1:**
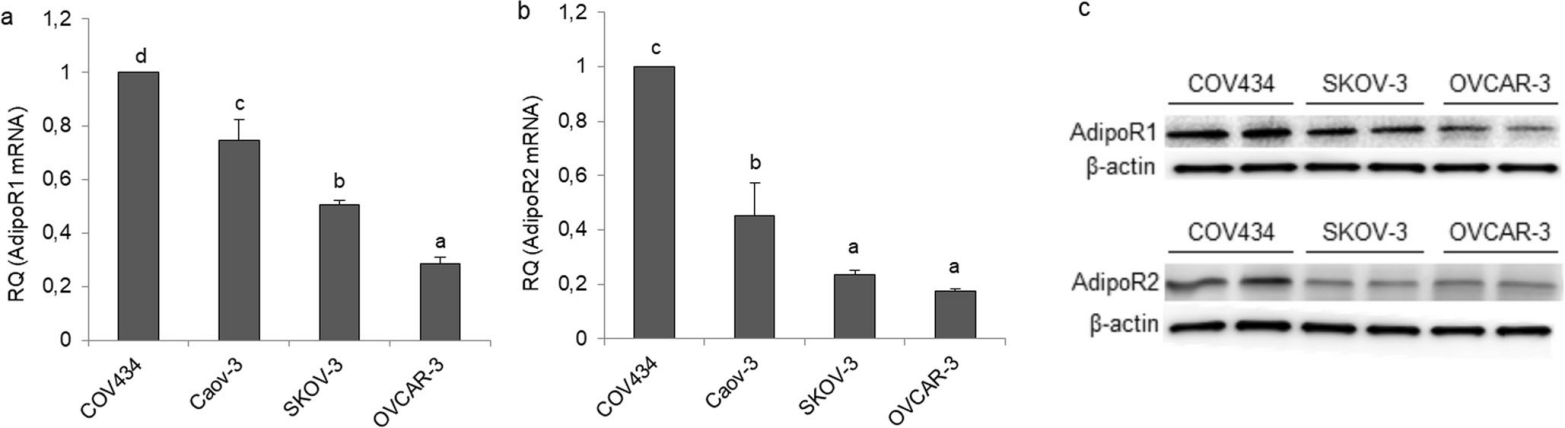
Basal mRNA and protein expression of adiponectin receptors (AdipoR1 and AdipoR2) in various cancer cell lines. AdipoR1 mRNA (**a**), AdipoR2 mRNA (**b**), and protein expression (**c**) in a human ovarian granulosa tumor cell line (COV434 cells) and epithelial ovarian cancer cell lines (Caov-3, SKOV-3, and OVCAR-3). RQ, relative quantity. The mRNA levels of *ADIPOR1* and *ADIPOR2* in COV434 cells were set at 1.0. Statistically significant differences are indicated with different letters (a < b < c < d, *p* < 0.05)

Before investigating the effects of adiponectin on human epithelial ovarian cancer cells, we examined the expression of adiponectin and its receptors in epithelial ovarian cancer cell lines (OVCAR-3, SKOV-3, and Caov-3) and compared the mRNA levels with those in the granulosa tumor cell line (COV434). By real-time PCR analyses, all cancer cell lines failed to express adiponectin (data not shown). Both *ADIPOR1* and *ADIPOR2* receptors were expressed by all cell lines. The relative quantity (RQ) of each transcript (*ADIPOR1* and *ADIPOR2*) expressed by the granulosa tumor cell line (COV434) was arbitrarily set at 1. The basal *ADIPOR1* mRNA level was lower in the epithelial ovarian cancer cells (3.5-, 2-, and 1.3-fold in OVCAR-3, SKOV-3, and Caov-3, respectively) than in the granulosa tumor cell line (Fig. 1a; *p* < 0.05). Similarly, the basal *ADIPOR2* transcript level was lower in OVCAR-3, SKOV-3, and Caov-3 cells (6-, 4.3-, and 2.2-fold in OVCAR-3, SKOV-3, and Caov-3, respectively) than in COV434 cells (Fig. 1b; *p* < 0.05). The basal AdipoR1 and AdipoR2 protein levels were examined by Western blot analysis in COV434, OVCAR-3, and SKOV-3 cells, and the results mirrored those from real-time PCR analyses (Fig. 1c).

### Effects of Environmental Estrogens (BPA and Its Analogs) on the Expression of Adiponectin Receptors

**Fig. 2:**
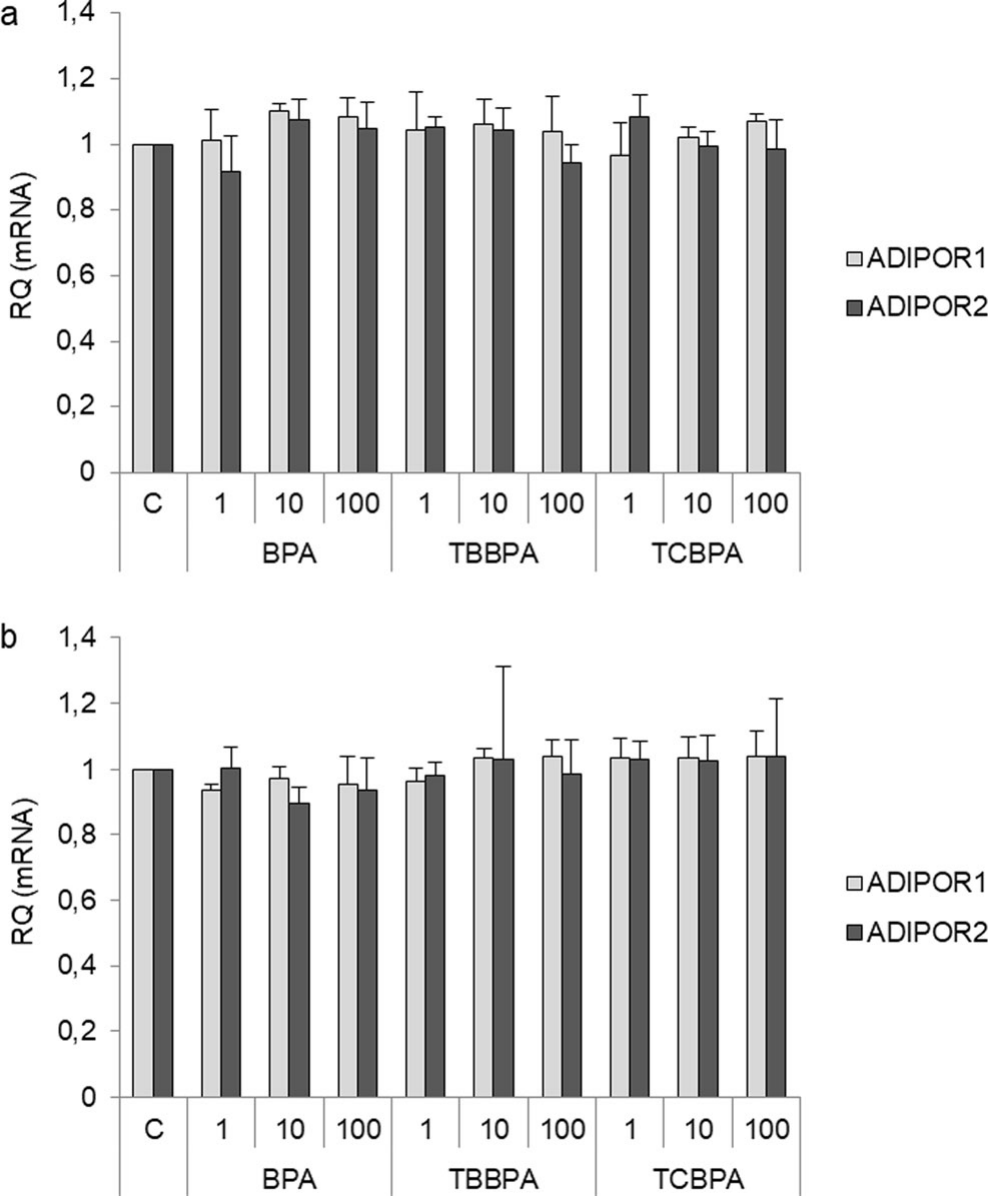
Effects of BPA, TBBPA, and TCBPA (1, 10, and 100 nM) on AdipoR1 and AdipoR2 mRNA expression in epithelial ovarian cancer cell lines (SKOV-3, **a**) and (OVCAR-3, **b**). *ADIPOR1* and *ADIPOR2* mRNA expression after exposing cells to the indicated compounds for 24 h. RQ, relative quantity; C, control. The mRNA levels of *ADIPOR1* and *ADIPOR2* in vehicle-treated cells were set at 1

A previous study reported that BPA regulates adiponectin production and secretion in 3T3-L1 adipocytes. For this reason, we assessed the effects of BPA, TBBPA, and TCBPA on the expression of AdipoR1 and AdipoR2 in SKOV-3 and OVCAR-3 cells by real-time PCR analysis. BPA, TBBPA, and TCBPA at all tested concentrations (1, 10, and 100 nM) had no effects on *ADIPOR1* and *ADIPOR2* expression in SKOV-3 (Fig. 2a) and OVCAR-3 cells (Fig. 2b).

### Effect of Adiponectin on OVCAR-3 and SKOV-3 Cell Proliferation

**Fig. 3:**
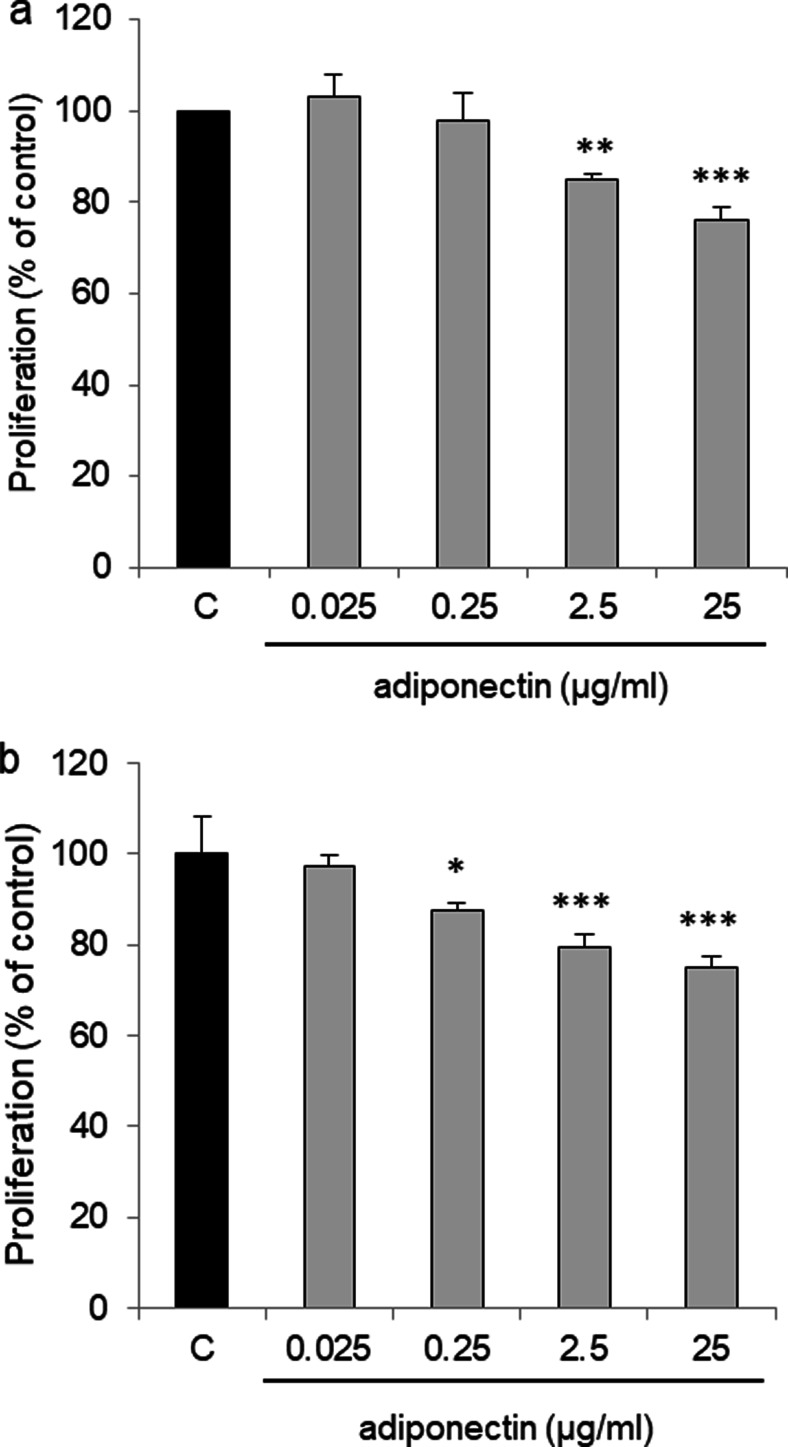
Effect of adiponectin (0.025, 0.25, 2.5, and 25 μg/ml) on the proliferation of OVCAR-3 (**a**) and SKOV-3 (**b**) cells after 48 h of treatment. C control. The proliferation rate in vehicle-treated cells was set at 100%. *p < 0.05, **p < 0.01, ***p < 0.001 compared with control cells

We examined the ability of adiponectin to affect OVCAR-3 and SKOV-3 cell proliferation. After 48 h of treatment, we observed a dose-dependent inhibition in the proliferation of both OVCAR-3 and SKOV-3 cells. Compared with that of control cells, there was a statistically significant decrease in proliferation after exposing OVCAR-3 cells to adiponectin at concentrations of 2.5 and 25 μg/ml (85 ± 1.5% and 76 ± 3% relative to the control, respectively) (Fig. 3a; *p* < 0.01, *p* < 0.001). Adiponectin at concentrations of 0.025 and 0.25 μg/ml had no effect on the proliferation of OVCAR-3 cells. Similarly, adiponectin decreased SKOV-3 cell proliferation dose-dependently (87 ± 1.7%, 79 ± 2.9% and 75 ± 2.5% at 0.25, 2.5, and 25 μg/ml relative to the control, respectively) (Fig. 3b; *p* < 0.05, *p* < 0.001).

Epithelial ovarian cancer cells differ from granulosa tumor cells in terms of their morphology and clinical behavior. We found that *ADIPOR1* and *ADIPOR2* expression was lowest in OVCAR-3 cells compared with that in the other cells. Moreover, our finding suggested that adiponectin inhibit OVCAR-3 and SKOV-3 cell proliferation in a similar manner. For this reason, OVCAR-3 cells were chosen as an in vitro model of serous epithelial ovarian cancer for further analysis.

### Effect of Adiponectin on Caspase-3 Activity and Cleaved PARP Protein Expression in OVCAR-3 Cells

**Fig. 4:**
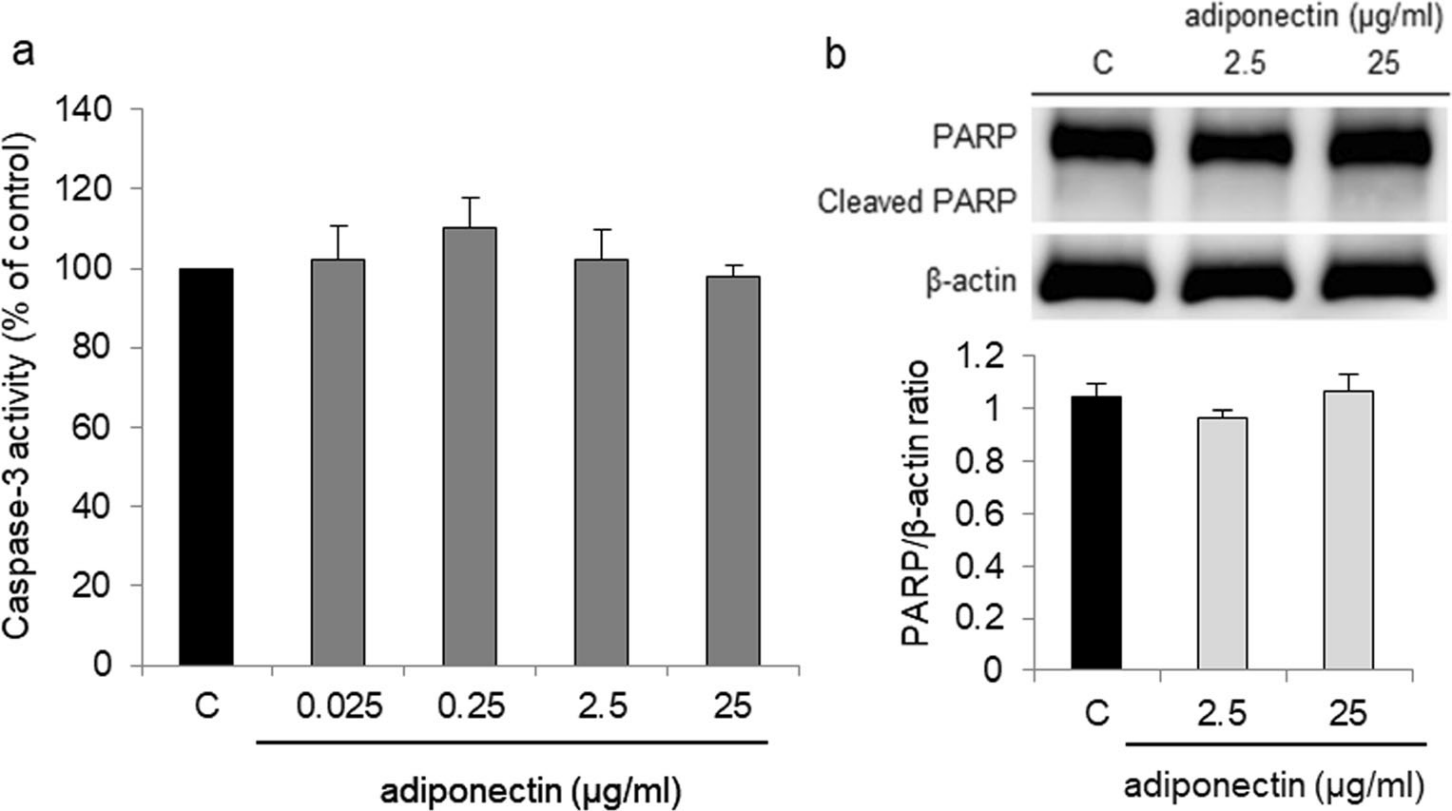
Effect of adiponectin (0.025, 0.25, 2.5, and 25 μg/ml) on caspase-3 activity (**a**) and cleaved PARP protein expression (**b**) in OVCAR-3 cells after 24 h of treatment. C control

In a parallel experiment that employed a caspase-3 activity assay, we evaluated the effect of adiponectin on caspase-3 activity and cleaved PARP protein expression in OVCAR-3 cells. Adiponectin at all tested concentrations had no effect on OVCAR-3 caspase-3 activity as well as cleaved PARP protein expression (Fig. 4).

### Effects of E2, IGF-1, and P4 on Adiponectin Reduces the Proliferation of Cancer Cells

**Fig. 5:**
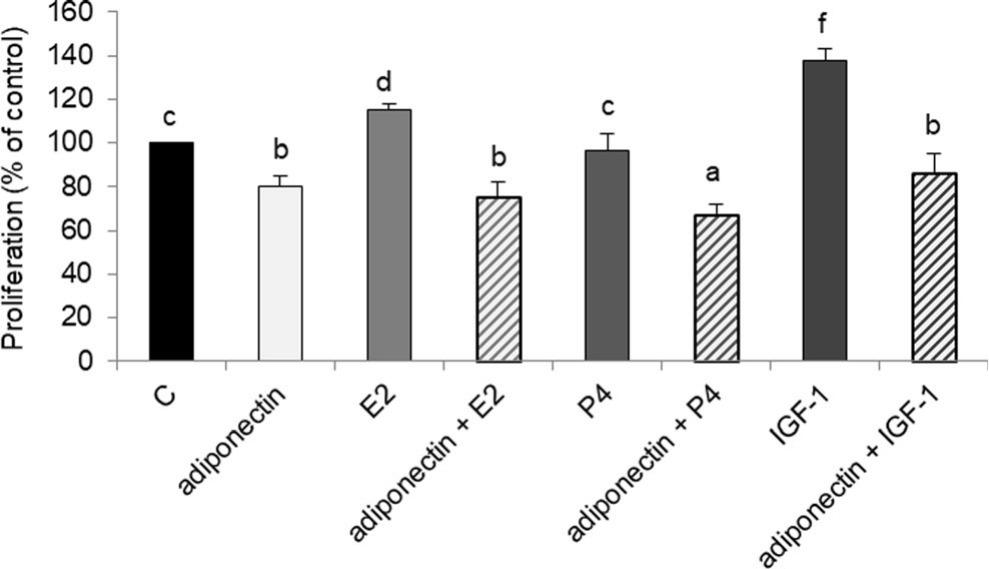
Effects of adiponectin (25 μg/ml) and E2 (10 nM), P4 (100 μM), or IGF-1 (100 ng/ml) on the proliferation of OVCAR-3 cells after 48 h of treatment. C control. Statistically significant differences are indicated with different letters (a < b < c < d < e < f, *p* < 0.05)

Previous studies reported E2, P4, and IGF-1 to affect ovarian cancer development, which prompted us to examine the effects of adiponectin and E2, P4, or IGF-1 on the proliferation of cancer cells. Consistent with previous results, we found that E2 and IGF-1 stimulated OVCAR-3 cell proliferation (115 ± 3% and 138 ± 5% relative to that of the control, respectively) (Fig. 5; *p* < 0.05). However, adiponectin reversed E2- and IGF-1-induced OVCAR-3 cell proliferation; the combined treatment of adiponectin and E2 or adiponectin and IGF-1 decreased cell proliferation to the basal adiponectin level after 48 h, (75 ± 7% and 86 ± 9%, respectively, vs 80 ± 5%), (Fig. 5; *p* < 0.05). Although P4 had no effect on OVCAR-3 cell proliferation, the combined treatment of adiponectin and P4 decreased cell proliferation to below the basal adiponectin level (67 ± 5% vs 80 ± 5%), (Fig. 5; *p* < 0.05).

### Effects of E2, P4, and IGF-1 on Adiponectin Receptor Expression

**Fig. 6:**
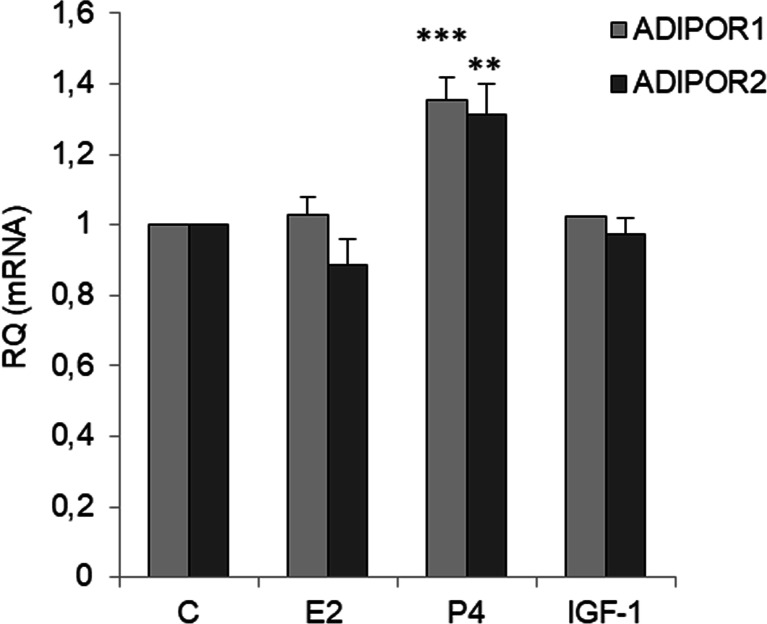
Effects of E2 (10 nM), P4 (100 μM), or IGF-1 (100 ng/ml) on *ADIPOR1* and *ADIPOR2* expression in OVCAR-3 cells. *ADIPOR1* and *ADIPOR2* mRNA expression after exposing cells to compounds for 24 h. RQ relative quantity, C control. The mRNA levels of *ADIPOR1* and *ADIPOR2* were set at 1. **p < 0.01, ***p < 0.001 compared with control cells

To investigate the mechanism by which adiponectin reduced E2- and IGF-1-induced cell proliferation, we measured the mRNA levels of AdipoR1 and AdipoR2 in OVCAR-3 cells after E2, IGF-1, and P4 treatment. As shown in Fig. 6, E2 and IGF-1 had no effect on the basal AdipoR1 and AdipoR2 levels. However, the levels of AdipoR1 and AdipoR2 were higher in OVCAR-3 cells cultured in the presence of P4 than in control cells (35 ± 6% for AdipoR1 and 31 ± 8% for AdipoR2 compared with the respective basal levels) (Fig. 6).

### Effects of Adiponectin on ERα, ERβ, PGR, and IGF1R Receptor Expression

**Fig. 7:**
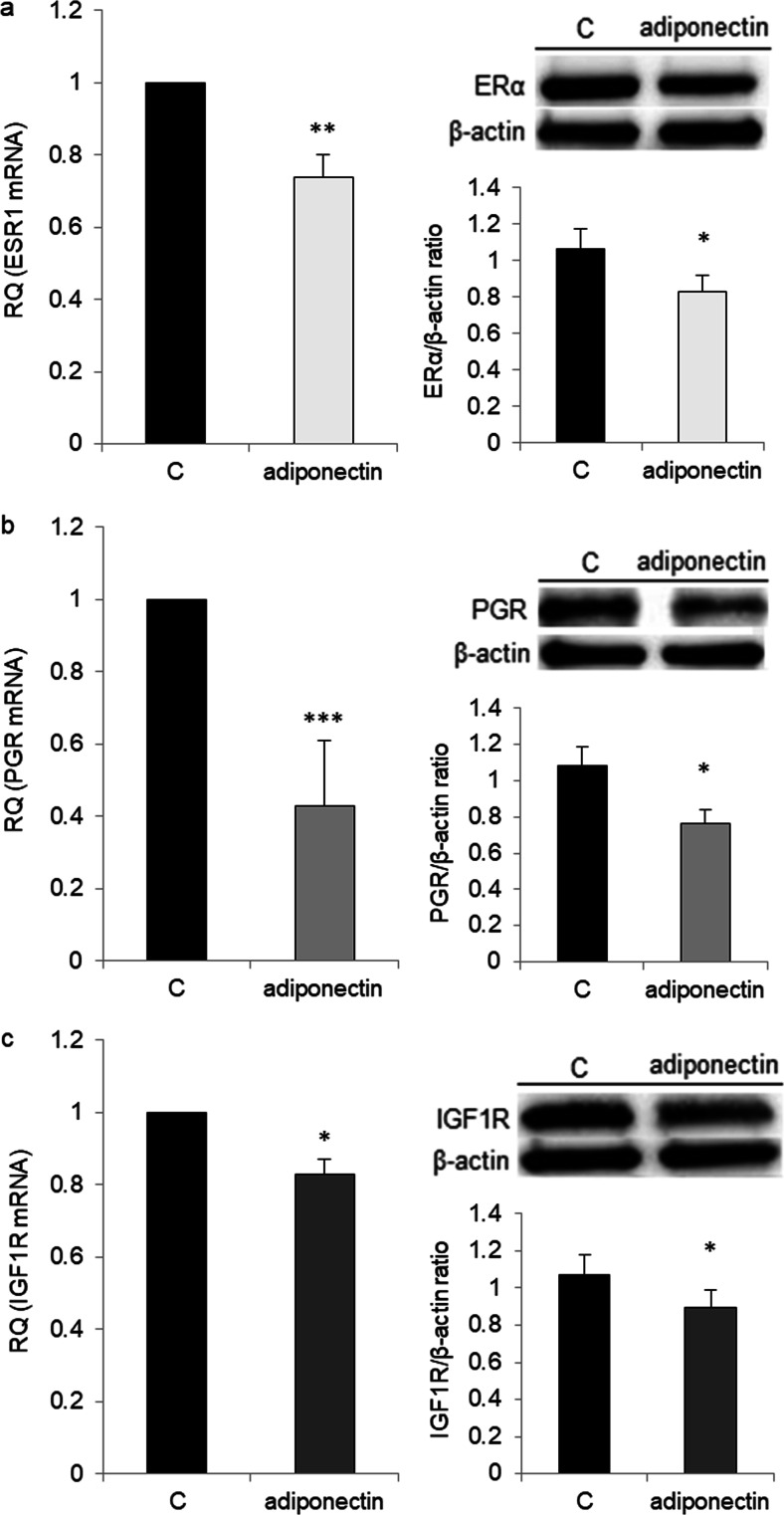
Effects of adiponectin (25 μg/ml) on the estrogen receptor (*ESR1*, **a**), progesterone receptor (*PGR*, **b**), and insulin-like growth factor type 1 receptor (*IGF1R*, **c**) mRNA at 24 h and protein at 48 h expression in OVCAR-3 cells. RQ relative quantity, C control. The control value was arbitrarily set at 1. *p < 0.05, **p < 0.01, ***p < 0.001 compared with control cells

Next, we examined whether adiponectin (25 μg/ml) could affect the mRNA expression of *ESR1*, *ESR2*, *IGF1R*, and *PGR* in OVCAR-3 cells. We observed no difference in *ESR2* expression between adiponectin-treated and control cells (data not shown). By contrast, the levels of *IGF1R*, *ESR1*, and *PGR* were significantly lower in adiponectin-treated cells than in control cells (0.83 ± 0.04, 0.74 ± 0.06, and 0.43 ± 0.1 vs 1 RQ, respectively). The ERα, PR, and IGF1R protein levels were examined by Western blot analysis, and the results mirrored those from real-time PCR analyses (Fig. 7).

## Discussion

The expression of AdipoR1 and AdipoR2 has been reported in a human granulosa tumor KGN cell line [14]. However, the majority (~ 90%) of malignant ovarian tumors are of epithelial cell origin, and only ~ 7% are classified as ovarian sex cord tumors, with the most common type being the granulosa tumor [2]. The expression of adiponectin and its receptors in epithelial ovarian cancer cells has not yet been reported. In the present study, we found that adiponectin was not expressed in the granulosa cell line, as well as epithelial ovarian cancer cell lines. These findings suggest that adiponectin can act on ovarian cancer cells in vivo only as an endocrine factor. Additionally, our results indicated that both AdipoR1 and AdipoR2 are expressed in various epithelial ovarian cancer cell lines, and that their expression in these cell lines was lower than in the granulosa tumor cell line (COV434). Our observations are consistent with those of Li et al. [13] and Tiwari et al. [29], who reported AdipoR1 expression in cancerous epithelial ovarian tissues. Moreover, Li et al. [13] illustrated that epithelial ovarian cancer patients with AdipoR1-positive expression survived longer than those with AdipoR1-negative expression. To our knowledge, there is no information on AdipoR2 expression in human ovarian cancer tissues. This study is the first to report a lower expression of AdipoR1 and AdipoR2 in epithelial ovarian cancer cells compared with granulosa tumor cells. This observation may partly explain why granulosa tumors are generally considered to have a better prognosis than epithelial ovarian tumors.

Second, we investigated whether BPA and its analogs could regulate the expression of AdipoR1 and AdipoR2 in epithelial ovarian cancer cells. We found that low nanomolar concentrations of BPA, as well as TBBPA and TCBPA, had no effects on the expression of both receptors in epithelial ovarian cancer cell lines (OVCAR-3 and SKOV-3). Previously, Hugo et al. [30] observed that BPA at 0.1 and 1 nM inhibited adiponectin secretion from human breast adipose tissue explants and abdominal subcutaneous explants. On the other hand, Kidani et al. [18] indicted that BPA and BPA-related chemicals, such as bisphenol F, bisphenol E, and bisphenol B, decreased adiponectin secretion in 3T3-L1 adipocytes. However, there is no information on the effects of BPA, TBBPA, or TCBPA on AdipoR1 and AdipoR2 expression in human ovarian cells.

Because the effects of adiponectin on epithelial ovarian cancer cells are unknown, we examined whether adiponectin at concentrations reported in the serum and lower can affect OVCAR-3 and SKOV-3 cell proliferation. We found that adiponectin inhibited the growth of both cell lines. Similar results have been observed in various breast cancer cell lines, including MCF-7 [10], MDA-MB-231 [11, 31], and T47D [11]. The anti-proliferative effects of adiponectin in breast cancer cells are sometimes [31], but not always [11, 32], associated with apoptosis. Considering this, we analyzed the effect of the adiponectin on ovarian cancer cell apoptosis. We observed that adiponectin had no effect on OVCAR-3 apoptosis. These data indicated that adiponectin decreased epithelial ovarian cancer cell proliferation, and that this effect was independent of apoptosis.

Next, we investigated whether ovarian hormones and a growth factor (E2, P4, and IGF-1) could affect adiponectin activity in epithelial ovarian cancer cells. We demonstrated the antagonistic effect of adiponectin on E2- and IGF-1-induced epithelial ovarian cancer cell proliferation. Consistent with previous data, we observed mitogenic activity after treatment of epithelial ovarian cancer cells with E2 [22, 33] and IGF-1 [34]. In the present study, proliferation was reduced to the basal adiponectin level in cells exposed to adiponectin and E2 or adiponectin and IGF-1. These results are consistent with those of Dieudonne et al. [10], who reported that adiponectin suppressed the proliferation of E2-treated breast cancer MCF-7 cells. However, there is no information on the effects of adiponectin on IGF-1-induced proliferation in ovarian cancer cells.

To gain insight into the mechanism underlying the antagonistic effects of adiponectin on E2- and IGF1-induced cell proliferation, we examined AdipoR1 and AdipoR2 expression after E2 and IGF-1 treatment and ERα, ERβ, and IGF1R expression after adiponectin treatment. Similar to exogenous estrogens, such as BPA, and endogenous estrogens, we found that E2 had no effect on AdipoR1 and AdipoR2 expression. We also found that IGF-1 did not affect AdipoR1 and AdipoR2 expression. However, adiponectin reduced the expression of ERα and IGF1R, but not that of ERβ, in epithelial ovarian cancer cells. In contrast to ERα, ERβ activates anti-proliferative pathways in ovarian cancer cells [35]. There is also an inverse correlation between ERβ expression and ovarian tumor progression [36, 37]. Results from this study demonstrated that adiponectin changes the ERα/β ratio by decreasing ERα expression in epithelial ovarian cancer cells. Similar to our observations, Jarde et al. [38] noted that adiponectin downregulated ERα gene expression in the MCF-7 breast cancer cell line. However, anti-proliferative adiponectin effects have been reported for ER-positive [10] and ER-negative [31] breast cancer cell lines, suggesting that the effects of adiponectin are not due to interactions with the estrogen pathway. Indeed, our results indicated that adiponectin not only interacts with the estrogen pathway, but also with the IGF-1 pathway.

We also found that P4 had no effect on the proliferation of OVCAR-3 cells. Murdoch et al. [39] arrived at a similar conclusion after performing experiments on P4-treated OVCAR-3 and SKOV-3 cells. Interestingly, we observed that co-treatment with adiponectin and P4 potentiated the effects of adiponectin by reducing cell proliferation to below the basal adiponectin level. We also found that P4 upregulated AdipoR1 and AdipoR2 expression, whereas adiponectin downregulated *PGR* expression. Thus, the potentiation of the effects of adiponectin by P4 enabled us to investigate the responsiveness of different cancer cell lines to the hormone. On the other hand, the decreased *PGR* expression altered the insensitivity of various epithelial ovarian cancer cells to the anti-proliferative properties of P4 [40]. Our results may be partly explained by interactions between P4 and leptin pathways. It is well documented that leptin, an adipocyte-secreted hormone, has opposite effects on the proliferation of cancer cells [41]. For example, Jardé et al. [38] demonstrated that leptin upregulated PR mRNA expression in MCF-7 cells. Moreover, P4 inhibited leptin receptor gene expression in the human endometrium [42]. Our results, together with those of previous studies, support the contention that the effects of adiponectin on cancer cells are opposite to those of leptin.

Taken together, these findings indicate that adiponectin represses the proliferation of epithelial ovarian cancer cells and reverses the stimulatory effects of E2 and IGF-1 on cell proliferation by downregulating the expression of their receptors. In addition, P4 increased the sensitivity of cancer cells to adiponectin by upregulating the expression of it receptors. These results also suggest interactions between adiponectin and various ovarian steroid hormone and growth factor pathways in ovarian cancer cells.
